# Fusing Carbocycles of Inequivalent Ring Size to a Bis(imino)pyridine-Iron Ethylene Polymerization Catalyst: Distinctive Effects on Activity, PE Molecular Weight, and Dispersity

**DOI:** 10.34133/2019/9426063

**Published:** 2019-10-16

**Authors:** Zheng Wang, Gregory A. Solan, Yanping Ma, Qingbin Liu, Tongling Liang, Wen-Hua Sun

**Affiliations:** ^1^Key Laboratory of Engineering Plastics and Beijing National Laboratory for Molecular Science, Institute of Chemistry, Chinese Academy of Sciences, Beijing 100190, China; ^2^CAS Research/Education Center for Excellence in Molecular Sciences, University of Chinese Academy of Sciences, Beijing 100049, China; ^3^Department of Chemistry, University of Leicester, University Road, Leicester LE1 7RH, UK; ^4^College of Chemistry and Material Science, Hebei Normal University, Shijiazhuang 050024, China; ^5^State Key Laboratory for Oxo Synthesis and Selective Oxidation, Lanzhou Institute of Chemical Physics, Chinese Academy of Sciences, Lanzhou 730000, China

## Abstract

The 4,6-bis(arylimino)-1,2,3,7,8,9,10-heptahydrocyclohepta[*b*]quinoline-iron(II) chlorides (aryl = 2,6-Me_2_C_6_H_3_**Fe1**; 2,6-Et_2_C_6_H_3_**Fe2**; 2,6-*i*-Pr_2_C_6_H_3_**Fe3**; 2,4,6-Me_3_C_6_H_2_**Fe4**; and 2,6-Et_2_-4-Me_2_C_6_H_2_**Fe5**) have been prepared in good yield by a straightforward one-pot reaction of 2,3,7,8,9,10-hexahydro-1H-cyclohepta[*b*]quinoline-4,6-dione, FeCl_2_·4H_2_O, and the appropriate aniline in acetic acid. All ferrous complexes have been characterized by elemental analysis and FT-IR spectroscopy. In addition, the structure of **Fe3** has been determined by single crystal X-ray diffraction, which showed the iron center to adopt a distorted square pyramidal geometry with the saturated sections of the fused six- and seven-membered carbocycles to be *cis*-configured. In combination with either MAO or MMAO, **Fe1**–**Fe5** exhibited exceptionally high activities for ethylene polymerization (up to 15.86 × 10^6^ g(PE) mol^−1^ (Fe) h^−1^ at 40°C (MMAO) and 9.60 × 10^6^ g(PE) mol^−1^ (Fe) h^−1^ at 60°C (MAO)) and produced highly linear polyethylene (HLPE, *T*_m_ ≥ 128°C) with a wide range in molecular weights; in general, the MMAO-promoted polymerizations were more active. Irrespective of the cocatalyst employed, the 2,6-Me_2_-substituted **Fe1** and **Fe4** proved the most active while the more sterically hindered 2,6-*i*-Pr_2_**Fe3** the least but afforded the highest molecular weight polyethylene (*M*_w__:_ 65.6–72.6 kg mol^−1^). Multinuclear NMR spectroscopic analysis of the polymer formed using **Fe4**/MMAO at 40°C showed a preference for fully saturated chain ends with a broad bimodal distribution a feature of the GPC trace (*M*_w_/*M*_n_ = 13.4). By contrast, using **Fe4**/MAO at 60°C a vinyl-terminated polymer of lower molecular weight (*M*_w_ = 14.2 kg mol^−1^) was identified that exhibited a unimodal distribution (*M*_w_/*M*_n_ = 3.8). Moreover, the amount of aluminoxane cocatalyst employed, temperature, and run time were also found to be influential on the modality of the polymer.

## 1. Introduction

**Figure 1:**
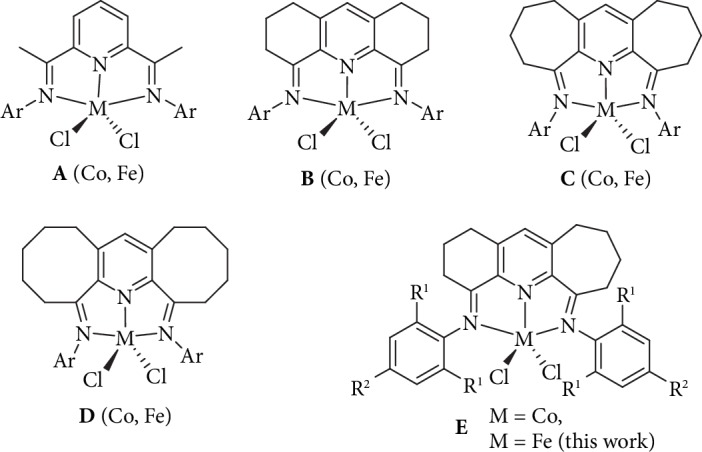
Bis(imino)pyridine-metal(II) chlorides **A** and their doubly fused derivatives **B**–**E** (metal = iron and cobalt).

The outstanding productivities attainable by bis(arylimino)pyridine-iron and bis(arylimino)pyridine-cobalt (pre)catalysts (**A**, Figure 1) for the polymerization of ethylene, initially reported over twenty years ago [1–4], have spurred a myriad of academic and industrial research disclosures [5–9]. Through systematic variation of the steric and electronic properties of the ligand frame and in particular to the N-aryl groups, catalysts capable of generating highly sought-after materials such as *α*-olefins, linear PE waxes, and high-density polyethylene (HDPE) are all accessible [2, 6, 10]. Moreover, such targeted ligand manipulation has seen remarkable improvements to the temperature stability of the catalyst itself [5–9, 11–14], a limitation often levelled against the first-generation catalysts [4, 6]. Elsewhere, other types of neutral *N,N,N*-ligand skeleton such as *N*-[(pyridin-2-yl)-methylene]-8-amino-quinolines [15], 2-benzimidazolyl-6-imino-pyridines [16–18], 2,8-bis(imino)quinolines [19], and 2-imino-1,10-phenanthrolines [20, 21] have witnessed some important developments [6–8]. From a commercial viewpoint, the successful implementation of a 500-ton scale pilot process for the production of linear *α*-olefins (LAOs) in China that makes use of a 2-imino-1,10-phenanthroline-iron catalyst highlights the enormous potential of this homogeneous technology [6, 8, 20, 21].

As an alternative strategy in bis(imino)pyridine ligand design, our group has recently explored the fusion of carbocycles to the central pyridine unit in (Figure 1) as a means to form well-defined Fe-/Co-based complexes bearing singly [22–30] and doubly fused derivatives (e.g., **B**, **C**, **D**, and **E** in Figure 1) [31–38]. Indeed, the fused ring size has been shown to be pivotal to the catalytic activity and polymeric properties [6, 36–38]. Of particular note, iron-containing **B** [31], **C** [32], and **D** [36] can produce strictly linear polyethylenes with a wide range of molecular weights, end-group types, and activities (up to 10^7^ g(PE) mol^−1^ (Fe) h^−1^) [6, 36]. For example, six-membered **B** produced the lowest molecular weight polymer (8 kg mol^−1^) [31], while seven- and eight-membered **C** and **D** showed a predilection towards higher molecular weight vinyl-polyethylenes (up to 188 kg mol^−1^) [32, 36]. In light of these performance differences, it is probable that the ring size impacts on properties such as the ring flexibility/tension and the overall steric properties of the chelating ligand which in turn influences the propagation and chain transfer steps of polymerization [6]. As a more recent development, we have demonstrated that cobalt-containing **E** (Figure 1), incorporating both six- and seven-membered carbocycles, not only showed the highest catalytic activity of the cobalt-containing **A**-**E** series but also generated valuable vinyl-terminated PE waxes with narrow molecular weight distributions [38]. Significantly, such low molecular weight polymers provide promising raw materials for the production of functional polymers, copolymers, and coating materials [32, 38].

Given the higher catalytic performance generally achievable for iron over cobalt in ethylene polymerization, [6–8] we now disclose five examples of iron-containing **E** (Figure 1) that differ in the steric (R^1^ = Me, Et, *i*-Pr) and electronic (R^2^ = H or Me) properties of their N-aryl groups. Their relative performance as precatalysts in ethylene polymerization is then evaluated using two types of aluminoxane cocatalyst; optimization studies concerned with the amount of aluminoxane cocatalyst employed, run temperature, time, and pressure are also highlighted. Moreover, key comparisons of **E** with the parent bis(imino)pyridine-iron **A** and its symmetrically fused derivatives **B**, **C**, and **D** are made in terms of catalytic performance as well as the properties of the polymer. In addition, full details of the preparation and characterization for the new iron(II) complexes are presented.

## 2. Results and Discussion

### 2.1. Synthesis and Characterization of the Iron(II) Complexes

**Scheme 1:**
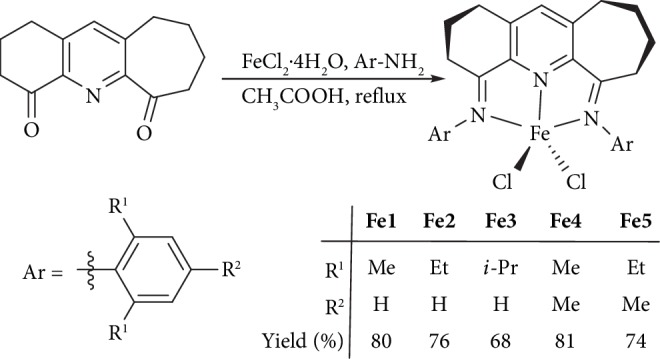
One-pot route to **Fe1**–**Fe5** from 2,3,7,8,9,10-hexahydro-1H-cyclohepta[*b*]quinoline-4,6-dione.

Treatment of 2,3,7,8,9,10-hexahydro-1H-cyclohepta[*b*]quinoline-4,6-dione [38] with FeCl_2_·4H_2_O and four equivalents of the corresponding aniline in acetic acid at reflux for 12 hours gave, on work-up, the 4,6-bis(arylimino)-1,2,3,7,8,9,10-heptahydrocyclohepta[*b*]quinoline-iron(II) chlorides (aryl = 2,6-Me_2_C_5_H_3_**Fe1**; 2,6-Et_2_C_5_H_3_**Fe2**; 2,6-*i*-Pr_2_C_5_H_3_**Fe3**; 2,4,6-Me_3_C_5_H_2_**Fe4**; and 2,6-Et_2_-4-MeC_5_H_2_**Fe5**), in good yields (68–81%) (Scheme 1). Such a one-pot template approach [31, 32, 36–38] was considered necessary as the free 4,6-bis(arylimino)-1,2,3,7,8,9,10-heptahydrocyclohepta[*b*]quinolines were not favorable to isolation. All new complexes have been characterized by FT-IR spectroscopy and elemental analysis. In addition, a crystal of **Fe3** was the subject of a single crystal X-ray diffraction study.

**Figure 2:**
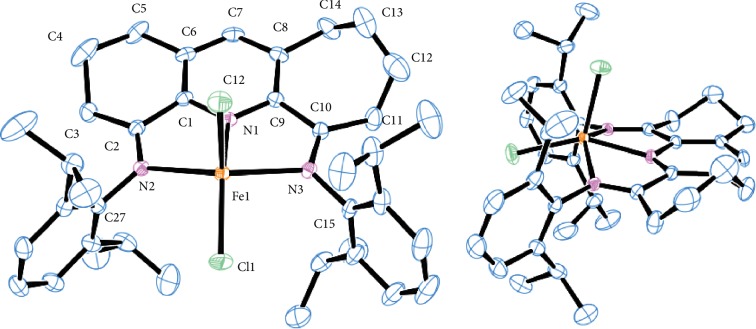
ORTEP representations of **Fe3** at the 30% probability level. Hydrogen atoms have been omitted for clarity.

Single crystals of **Fe3** suitable for the X-ray determination were grown under an atmosphere of nitrogen by the slow diffusion of Et_2_O into a CH_2_Cl_2_ solution of the complex maintained at room temperature. A view of **Fe3** is depicted in Figure 2; selected bond distances/angles are collected in Table 1. The structure of **Fe3** consists of a single iron center surrounded by three nitrogen atoms belonging to the 4,6-bis(2,6-diisopropylphenylimino)-1,2,3,7,8,9,10-heptahydrocyclohepta[*b*]quinoline and two terminal chlorides to afford a geometry best termed distorted square pyramidal. Specifically, the nitrogen donor atoms (N1, N2, and N3) of the chelating ligand together with Cl1 form the square base of the pyramid with Cl2 occupying the apical position; related arrangements have previously been seen for their iron-based counterparts **B**–**D** (Figure 1) [31, 32, 36]. The iron atom in **Fe3** lies at a distance of 0.562 Å above the square base, which is slightly shorter than in **D** (0.596 Å) [36] and 2-(1-(arylimino)ethyl)-8-arylimino-5,6,7-trihydroquinoline-iron(II) chloride (0.583 Å) [22], while longer than that found in iron complexes bound by 2-(arylimino)-9-arylimino-5,6,7,8-tetrahydro-cycloheptapyridines (0.442 and 0.320 Å) [26, 27]. Within the *N,N,N*-Fe unit, the Fe–N_pyridine_ bond distance of 2.060(1) Å is noticeably shorter than the exterior Fe–N_imine_ bond lengths of 2.216(1) Å, while the N1–Fe1–N2 and N1–Fe1–N3 angles of 74.17(7)° and 73.10(7)° are comparable with previous work [22, 26, 27, 32, 36]. Moreover, the two Fe–N_imine_ bond lengths in **Fe3** are slightly shorter when compared with those in **C** (2.313(4)-2.320(4) Å) [32] and **D** (2.261(2)-2.272(2) Å) [36] but similar to those observed in iron(II)-containing 2-(arylimino)-9-arylimino-5,6,7,8-tetrahydro-cycloheptapyridines (2.210(8) and 2.212(9) Å) [26]. Similarly, the Fe–N_pyridine_ distance in **Fe3** is shorter than that found in **A** [4], **B** [31], **C** [32], and **D** [36] (range: 2.080(4)–2.189(6) Å) but related to those observed in the 2-(1-(2,6-diethylphenylimino)ethyl)-8-arylimino-5,6,7-trihydroquinoline-iron(II) chlorides (2.069(3) Å) [22]. Examination of the N1–C1–C2–N2 (6.40°) and N1–C9–C10–N3 (-6.93°) torsion angles highlights the deviation from coplanarity between the pyridine ring and the adjacent imine vectors; a related distorted arrangement of the *N,N,N*-ligand has been noted in its cobalt analogues [38]. The C3-C4-C5 and C11-C12-C13-C14 sections of the two fused carbocyles are puckered as a consequence of the sp^3^-hybridization of these carbon atoms leading to a *cis* configuration in which both saturated sections fold towards apical Cl2. The N-aryl rings are inclined at angles of 88.93° (N2_6-membered_) and 77.78° (N3_7-membered_) with respect to their neighboring imine vectors, which can be justified in terms of the different steric properties exerted by the 6- and 7-membered rings. Collectively, it would appear that the fused 6- and 7-membered rings in **Fe3** have caused some structural reorganization within the *N,N,N*-ligand which in turn influences the coordination sphere of the complex.

The FT-IR spectra of **Fe1**–**Fe5** revealed stretching frequencies in the range 1604–1614 cm^−1^ that are quite typical of bound N-imine groups; no absorptions corresponding to the free diketone were detectable [36–39]. Further support for the structural identity of **Fe1**–**Fe5** was provided by the microanalytical data which were in complete agreement with elemental compositions for complexes of general formula (*N,N,N*)FeCl_2_.

### 2.2. Ethylene Polymerization

To investigate the aptitude of **Fe1**–**Fe5** to act as precatalysts for ethylene polymerization, MAO and MMAO were selected as cocatalysts to allow two parallel studies to be performed. Both these types of aluminoxane have been previously shown as among the most effective for precatalyst activation in iron-based polymerization catalysis [4, 6, 11, 12, 26, 27, 31, 32, 36].

#### 2.2.1. Catalytic Evaluation of **Fe1**–**Fe5** Using MMAO

To establish the optimum reaction conditions for these MMAO-activated polymerizations, **Fe4** was chosen as the precatalyst for initial assessment and the amount of aluminoxane cocatalyst (Al : Fe molar ratio), temperature, and run time systematically varied with the ethylene pressure set at either 1, 5, or 10 atm.

Firstly, the runs were performed at 1 atm C_2_H_4_ and the effect of Al : Fe molar ratio on the performance of **Fe4**/MMAO examined (runs 1-5, Table 2). With the reaction temperature kept at 20°C, the tests were undertaken using different Al : Fe ratios of 1000, 1500, 2000, 2500, and 3000; the optimum activity of 1.82 × 10^6^ g(PE) mol^–1^ (Fe) h^–1^ was observed at a value of 2500 (run 4, Table 3). The polyethylenes for all five runs were of low molecular weight falling in the range 5.8–15.7 kg mol^−1^; there was no evidence for any short chain oligomers (e.g., from C_4_ to C_32_). It was also noted that the lowest molecular weight polymer within this range corresponded to the polymerizations conducted with higher Al : Fe molar ratios, a finding that can be linked to the higher rate of chain transfer from the iron active species to aluminum on increasing the amount of alkyl aluminum reagent [26, 27, 32, 36–38, 40, 41]. The influence of temperature was then investigated with the Al : Fe molar ratio retained at 2500. By varying the test temperature from 10 to 60°C (runs 4 and 6–10, Table 2), a peak activity of 1.82 × 10^6^ g(PE) mol^–1^ (Fe) h^–1^ was observed at 20°C. It was also evident that the molecular weight of the polyethylene gradually decreased from 9.5 to 1.2 kg mol^–1^ as the temperature was raised which can be credited to increased chain transfer at higher temperature [4, 22, 26, 27, 32–38]. Meanwhile, the molecular weight distribution narrowed as the temperature was raised (*M*_w_/*M*_n_: from 13.2 to 2.2), an observation also noted at higher ethylene pressure (*vide infra*) and elsewhere [4, 32, 36].

**Figure 3:**
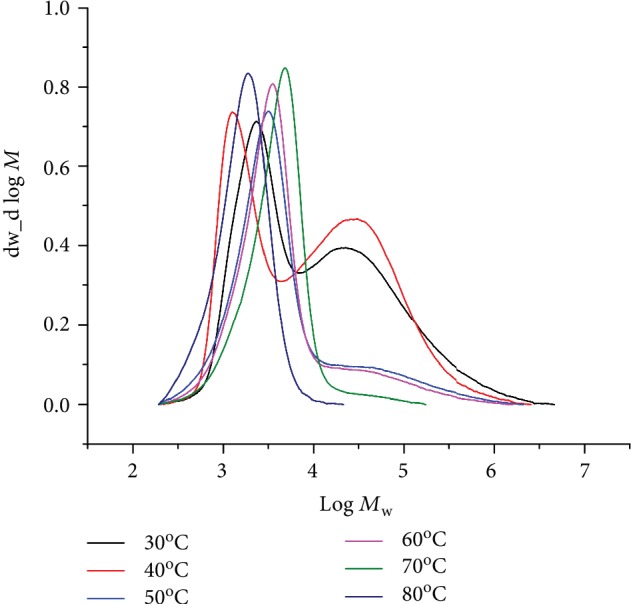
GPC traces for the PEs produced using **Fe4**/MMAO at different reaction temperatures (10 atm C_2_H_4_, Al : Fe ratio = 2500, and 30 min; runs 1–6, Table 3).

Secondly, **Fe4**/MMAO was also screened at 10 atm C_2_H_4_; the experimental findings are compiled in Table 3. Bearing in mind the temperature/activity correlations seen at 1 atm C_2_H_4_, a similar study was performed at 10 atm with the Al : Fe molar ratio kept at 2500. On increasing the temperature from 30 to 80°C (runs 1-6, Table 3), the optimum activity of 15.15 × 10^6^ g(PE) mol^−1^ (Fe) h^−1^ was observed at 40°C. Notably, only modest reductions were evident at either 30 or 50°C, with all values falling at the 10^7^ g(PE) mol^−1^ (Fe) h^−1^ level. Indeed, only with the temperature above 60°C did the activity start to significantly drop, with a relatively low value of 4.49 × 10^6^ g(PE) mol^−1^ (Fe) h^−1^ observable at 80°C (run 6, Table 3). As shown in Figure 3, the molecular weights of the polyethylenes decreased from 59.9 to 1.9 kg mol^–1^ as the temperature was increased from 30 to 80°C, while the molecular weight distributions ranged from bimodal (≤40°C), with two *M*_pk_ peaks (peak 1 and peak 2) clearly visible in their GPC traces (Figure 3), to unimodal (≥50°C) [36]. Moreover, the molecular weight distributions progressively narrowed as the temperature was raised (*M*_w_/*M*_n_: from 16.0 to 1.5). To account for the modality variations, it would seem likely that two different chain transfer pathways were occurring at temperatures of ≤40°C (e.g., *β*-H elimination and transfer to aluminum) while at ≥50°C one type of chain transfer was prevalent [11, 13, 32, 36, 37].

**Figure 4:**
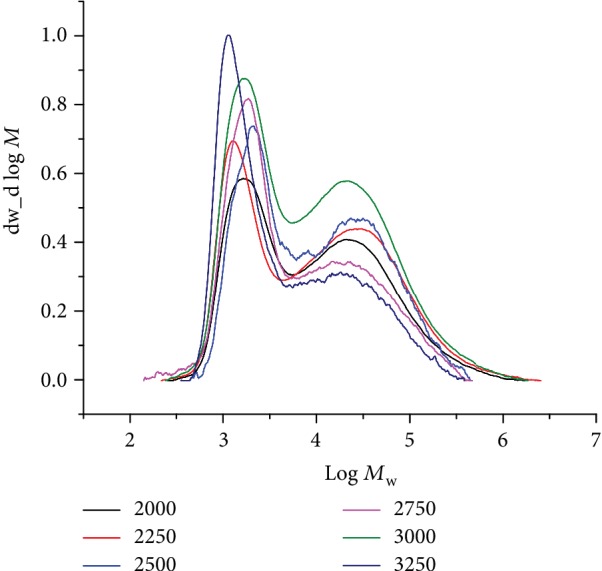
GPC traces for the PEs produced using **Fe4**/MMAO with various Al : Fe ratios (10 atm C_2_H_4_, 40°C, and 30 min; runs 2 and 7–11, Table 3).

With the temperature held at 40°C, the polymerization tests were then executed using five different Al : Fe ratios (2000, 2250, 2500, 2750, 3000, and 3250). Inspection of the data indicates that there were only modest effects on the activity across this range in Al : Fe ratios (runs 2 and 7–11, Table 3). Nevertheless, the highest value of 15.15 × 10^6^ g(PE) mol^−1^ (Fe) h^−1^ was observed at a ratio of 2500 (run 2, Table 3). On the other hand, the GPC traces indicated that the molecular weight of the polymers gradually decreased from 35.5 to 16.7 kg mol^−1^ on increasing the ratio from 2000 to 3250 (Figure 4). As noted at 1 atm C_2_H_4_, this finding can be ascribed to chain transfer from the active species to aluminum on increasing the amount of MMAO resulting in faster chain termination and lower molecular weight polymers [4, 26, 27, 32, 36, 40, 41]. A similar trend has been observed for their symmetrical comparators **B**, **C**, and **D** (Figure 1) [26, 27, 32, 36]. Notably, bimodal distributions (*M*_w_/*M*_n_: from 11.0 to 7.7) were again a characteristic of all these runs with two *M*_pk_ peaks (peaks 1 and 2) viewable in their GPC traces (Figure 4) with the higher molecular weight fraction progressively becoming the minor component with larger amounts of MMAO [26, 27, 36, 37].

To investigate the effect of the reaction time on the polymerization, the tests were conducted at run times of between 5 and 60 minutes (runs 2 and 12–15, Table 3) with the Al : Fe ratio fixed at 2500 and the temperature at 40°C. A maximum level of activity of 37.36 × 10^6^ g(PE) mol^−1^ (Fe) h^−1^ was attained after 5 minutes (run 12, Table 3), which by the 60-minute mark had noticeably lessened to 8.55 × 10^6^ g(PE) mol^−1^ (Fe) h^−1^ (run 15, Table 3). This would imply that the active species was rapidly produced following MMAO addition and then suffered gradual deactivation as the time elapsed [22, 24, 26, 32, 36, 40, 41]. In addition, the molecular weight of the polymers steadily increased over time (*M*_w_: from 5.8 to 72.9 kg mol^−1^) with broad bimodal distributions becoming a key feature of the GPC traces over longer test times; notably, the higher molecular weight fraction became the more significant one with more extended run times ([Supplementary-material supplementary-material-1]). On reduction of the ethylene pressure from 10 to 5 atm, the activity (8.52 × 10^6^ g(PE) mol^−1^ (Fe) h^−1^) dropped by nearly a half (run 16 vs. 3, Table 3). By comparison, at 1 atm C_2_H_4_, the lowest activity (1.82 × 10^6^ g(PE) mol^−^ (Fe) h^−1^, Table 2) was observed which can be credited to the lower ethylene concentration at lower pressure [22, 23, 26, 27, 32, 36–38].

Thirdly, to glean some information as to the effect imparted by the N-aryl groups on performance and polymer properties, **Fe1**–**Fe3** and **Fe5** were additionally screened for ethylene polymerization using the optimal conditions found for **Fe4**/MMAO (Al : Fe = 2500, *T* = 40°C, *t* = 30 min) (runs 17–20, Table 3). As a common observation, the five precatalysts exhibited very high activities (range: 8.50–15.86 × 10^6^ g(PE) mol^−1^ (Fe) h^−1^) and produced polyethylenes with broad bimodal distributions (*M*_w_/*M*_n_: from 13.4 to 24.7), which is in line with two chain transfer pathways being operational for all systems [26, 27, 32, 33, 36, 37]. In terms of the relative activity, this was found to fall in the order **Fe1** [2,6-di(Me)] > **Fe4** [2,4,6-tri(Me)] > **Fe2** [2,6-di(Et)] > **Fe5** [2,6-di(Et)-4-Me] > **Fe3** [2,6-di(*i*-Pr)], suggesting that the more bulky R^1^ substituents (e.g., **Fe3**, R = *i*-Pr) slowed down the coordination and insertion of ethylene [4, 32, 33, 36, 37, 42], while the least bulky ones (e.g., **Fe1** and **Fe4**, R = Me) promoted it. In terms of the *para*-substituent R^2^, it would appear that electron-donating groups are detrimental to activity (e.g., **Fe1** vs. **Fe4** and **Fe2** vs. **Fe5**). As a further point, the polyethylene obtained with the most hindered precatalyst, **Fe3** [2,6-di(*i*-Pr)], exhibited the highest molecular weight (65.6 kg mol^−1^, run 19, Table 3), which highlights the role of steric factors on influencing the chain transfer process [22, 23, 32, 36, 42].

**Figure 5:**
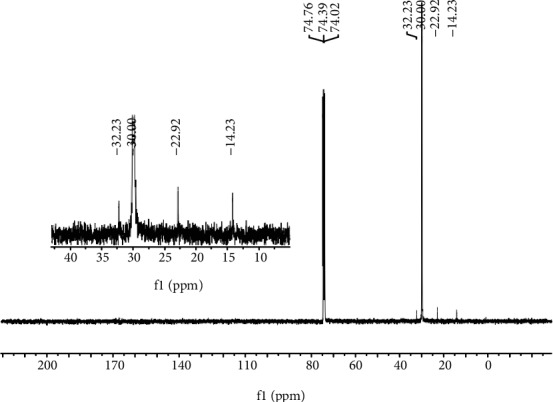
^13^C NMR spectrum of the polyethylene produced using **Fe4**/MMAO at 40°C; recorded in C_2_D_2_Cl_4_ at 135°C (run 2, Table 3).

**Figure 6:**
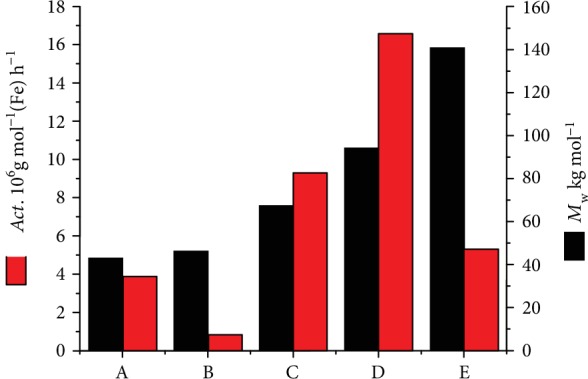
Comparative catalytic performance of **E**_Me2Ph_ (**Fe1**) with iron precatalysts **A**_Me2Ph_, [4] **B**_Me2Ph_ [31], **C**_Me2Ph_ [32], and **D**_Me2Ph_ [36] (Figure 1); all runs conducted using MMAO, *P*_C2H4_ = 10 atm and at 40°C.

In most cases, the *T*_m_ values of the polymers obtained using **Fe1**–**Fe5**/MMAO were above 128°C. As a representative sample, the polyethylene obtained using **Fe4**/MMAO at 40°C (*T*_m_ = 129.1°C, entry 2, Table 3) was characterized by ^13^C NMR spectroscopy. The spectrum, recorded in 1,1,2,2-tetrachloroethane-*d*_2_ (C_2_D_2_Cl_4_) at 135°C, revealed a high intensity peak around *δ* 30.00 which is indicative of a highly linear polyethylene (Figure 5) [6, 22, 32, 33, 36–38]. In addition, lower intensity resonances at *δ* 14.23, 22.92, and 32.23 were discernable that could be assigned to a *n*-propyl end-group (Figure 6) [11, 13, 32, 36–38, 43, 44]. By contrast, there was no evidence for signals corresponding to *i*-butyl end-groups, precluding chain transfer to Al(*i*-Bu)_3_ and its derivatives present in MMAO [11, 13, 32, 36], nor was there any detectable evidence for vinylic carbon resonances. Therefore, it would appear that the bimodal polymer generated using **Fe4**/MMAO (run 2, Table 3) contains substantial amounts of saturated chain ends formed through transfer to selectively AlMe_3_ and its derivatives present in MMAO [11, 36].

Finally, to allow a comparison of the current catalysts (**E**/MMAO) with previously reported iron systems (**A**–**D**, Figure 1), selected catalytic and polymer parameters for polymer samples prepared using **A**_Me2Ph_/MMAO, **B**_Me2Ph_/MMAO, **C**_Me2Ph_/MMAO, **D**_Me2Ph_/MMAO (Figure 1), and (**E**_Me2Ph_)/MMAO (where **E**_Me2Ph_ = **Fe1**) are displayed alongside each other in Figure 6 (see SI, Tables [Supplementary-material supplementary-material-1]–[Supplementary-material supplementary-material-1]). To maintain consistent conditions for the five catalysts, the polymerization tests for **A**_Me2Ph_/MMAO and **B**_Me2Ph_/MMAO had to be reperformed at 10 atm C_2_H_4_ (see SI, Tables [Supplementary-material supplementary-material-1] and [Supplementary-material supplementary-material-1]) as the original reports used either lower pressure (1.3 bar) or MAO as cocatalyst [4, 31]. All the MMAO-activated systems exhibited very high activities when the polymerization runs were performed at either 40°C or 50°C (up to 10^7^ g(PE) mol^−1^ (Fe) h^−1^), with their relative values at 40°C following in decreasing order **E**_Me2Ph_ > **D**_Me2Ph_ > **C**_Me2Ph_ > **B**_Me2Ph_~**A**_Me2Ph_. They also produced a range of different types of polymers from polyethylene waxes to high molecular weight polyethylene. Indeed, their molecular weights, as a function of the iron precatalyst, were found to decrease in the order **D**_Me2Ph_ > **C**_Me2Ph_ > **E**_Me2Ph_ > **A**_Me2Ph_ > **B**_Me2Ph_. Strikingly, **E**_Me2Ph_/MMAO was the most active catalyst (15.86 × 10^6^ g(PE) mol^−1^ (Fe) h^−1^ at 40°C, [Supplementary-material supplementary-material-1]) and formed polymer with a molecular weight in the midrange of the values. This trend in molecular weight indicates that the smaller the ring size of the carbocycle, the lower the molecular weight of the resultant polyethylene. As would be anticipated, the molecular weight of the polymer produced using the 6/7-membered **E**_Me2Ph_ lies in between that seen for its symmetrical fused-ring comparators **B**_Me2Ph_ (6/6) and **C**_Me2Ph_ (7/7). While steric effects imparted by the fused carbocycle are undoubtedly influential on the molecular weight, it would seem likely that other factors such as ring flexibility and chelation properties also play a role on affecting activity and molecular weight [6, 36, 38]. Pertaining to the dispersity of the polymers, the relatively inflexible (6/6) **B**_Me2Ph_/MMAO exhibited the narrowest distribution (*M*_w_/*M*_n_ = 4.5), while the somewhat more flexible (6/7) **E**_Me2Ph_/MMAO (*M*_w_/*M*_n_ = 10.8) was broader and the most flexible (8/8) **D**_Me2Ph_/MMAO displayed the broadest (*M*_w_/*M*_n_ = 28.6). Overall, these data not only highlight the importance of the fused ring size but also the effect of mixed rings on influencing catalytic performance, molecular weight, and dispersity [4, 6, 26, 27, 32, 36, 38].

#### 2.2.2. Catalytic Evaluation of **Fe1**–**Fe5** Using MAO

As with the **Fe**/MMAO study, **Fe4** was again initially screened this time in combination with MAO at 1 atm C_2_H_4_. With the temperature held at 20°C, the polymerization tests were performed using the different Al : Fe ratios, 1000, 1500, 2000, 2500, and 3000; the results are gathered in Table 4 (runs 1–5, Table 5). The best activity (6.53 × 10^5^ g(PE) mol^−1^ (Fe) h^−1^) was observed at an Al : Fe ratio of 2000. The molecular weights of the resultant polymers were found to decrease gradually from 85.4 to 27.6 kg mol^−1^ on raising the Al : Fe ratios from 1000 to 3000. As with the lower pressure runs undertaken using **Fe4**/MMAO, the polymers generated using **Fe4**/MAO at 1 atm C_2_H_4_ also displayed broad molecular weight distributions (*M*_w_/*M*_n_ = 13.7–20.6) over the range in molar ratios. Meanwhile, an investigation of the reaction temperature was conducted at 1 atm C_2_H_4_ with the Al : Fe ratio at 2000. By raising the temperature from 10 to 60°C (runs 3 and 6–10, Table 5), the topmost activity of 12.13 × 10^5^ g(PE) mol^−1^ (Fe) h^−1^ was observed at 30°C, while at 60°C only trace amounts of polymer were detected.

**Figure 7:**
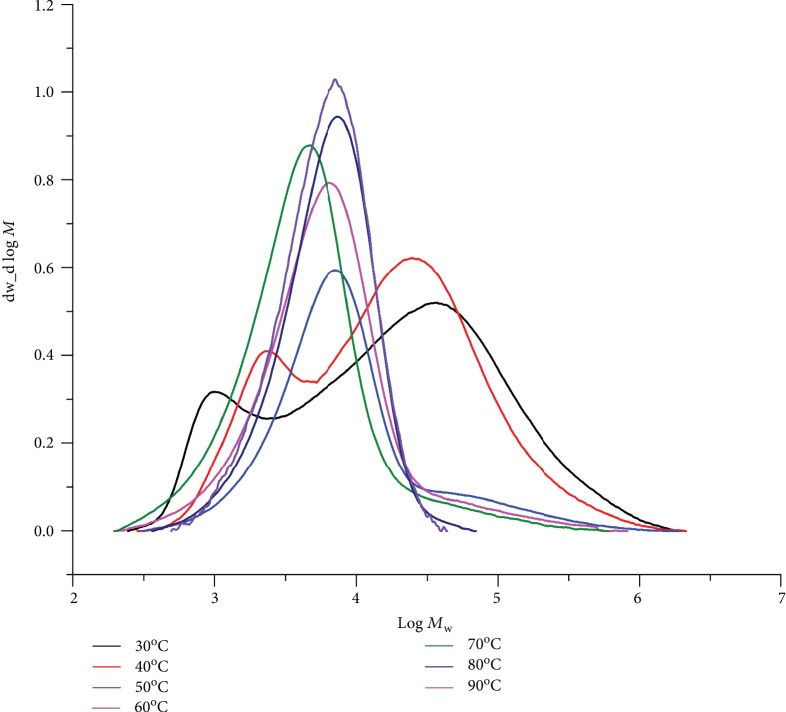
GPC traces for the PEs generated using **Fe4**/MAO at different reaction temperatures (10 atm C_2_H_4_, Al : Fe ratio = 2000, and 30 min; runs 1–7, Table 4).

Subsequently, the performance of **Fe4**/MAO at higher ethylene pressure was carried out; the results are tabulated in Table 4. The influence of reaction temperature was firstly explored at 10 atm C_2_H_4_ with an Al : Fe molar ratio of 2000. On increasing the reaction temperature from 30 to 90°C (runs 1-7, Table 4), the maximum activity of 9.28 × 10^6^ g(PE) mol^−1^ (Fe) h^−1^ was observed at 60°C which represents a higher optimum operating temperature to that seen with **Fe4**/MMAO; this finding highlights the improved thermal stability of the current catalyst. As borne out by their GPC traces (Figure 7), the molecular weights of the polyethylenes decreased from 64.2 to 7.5 kg mol^–1^ on elevating the temperature from 30 to 90°C, which as mentioned earlier can be ascribed to temperature-induced chain transfer [4, 26, 27, 32, 33, 36, 38, 40, 41]. As with **Fe4**/MMAO, the GPC traces obtained using **Fe4**/MAO over the 30–90°C range indicated the distributions to be bimodal-like at temperatures of ≤40°C, while at ≥50°C they become more unimodal (Figure 7) [36, 37].

**Figure 8:**
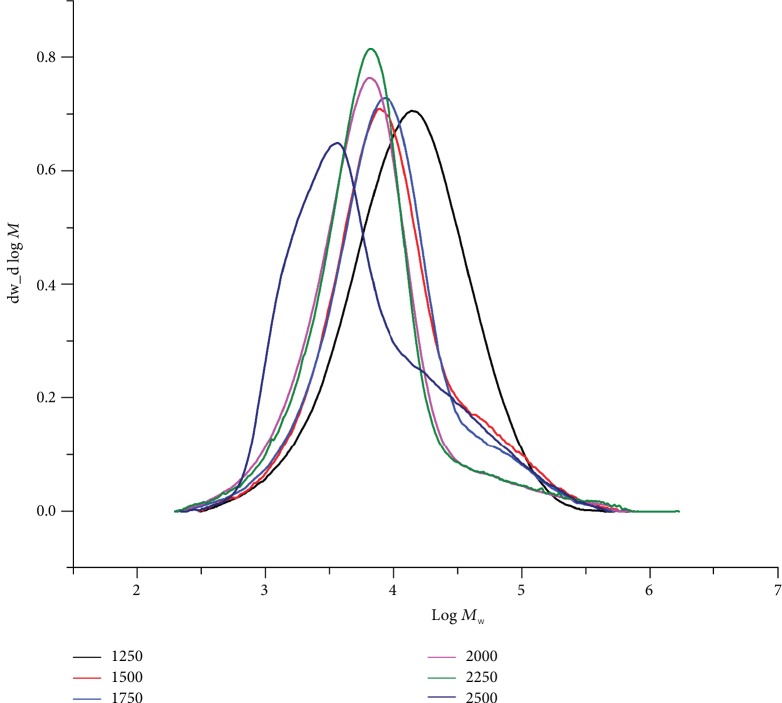
GPC traces for the PEs generated using **Fe4**/MAO with various Al : Fe ratios (10 atm C_2_H_4_, 60°C, and 30 min; runs 4 and 8–12, Table 4).

With the temperature at 60°C, the polymerization runs were carried out using different Al : Fe ratios of 1250, 1500, 1750, 2000, 2250, and 2500 (runs 4 and 8–12, Table 4). The results indicate little effect on the activity across this range in ratios with the highest value of 9.60 × 10^6^ g(PE) mol^−1^ (Fe) h^−1^ achievable with an Al : Fe ratio of 1500 (run 9, Table 4). On the other hand, the molecular weight of the polymeric materials was found to decrease gradually from 21.8 to 15.9 kg mol^−1^ on changing the ratio from 1250 to 2500 (Figure 8), on account of the more rapid chain transfer [4, 26, 27, 36, 37, 43–46]. In comparison with the **Fe4**/MMAO system, the polymers obtained using **Fe4**/MAO displayed a narrower molecular weight distribution over the range in molar ratios (*M*_w_/*M*_n_ range: 3.1–5.1).

**Figure 9:**
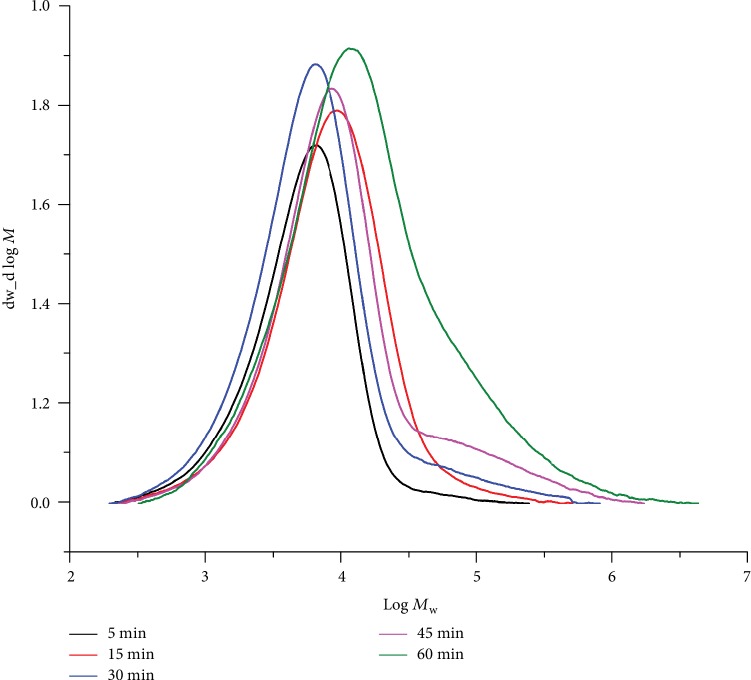
GPC traces for the PEs generated using **Fe4**/MAO at different reaction times (10 atm C_2_H_4_, 60°C, and Al : Fe ratio = 1500; runs 9 and 13–16, Table 4).

To facilitate an investigation of the catalytic lifetime of **Fe4**/MAO, the runs were performed over different reaction times from 5 to 60 minutes at 60°C and at an Al : Fe ratio of 1500 (runs 9 and 13–16, Table 4). The highest activity of 21.44 × 10^6^ g(PE) mol^−1^ (Fe) h^−1^ was observed after 5 minutes (run 13, Table 4) as was the case with **Fe4**/MMAO. The activity then gradually decreased to 5.39 × 10^6^ g(PE) mol^−1^ (Fe) h^−1^ after 60 minutes (run 16, Table 4) in agreement with gradual deactivation of the active species [22, 26, 27, 32, 33, 36–38, 40, 41]. In addition, the molecular weight of the polymers progressively increased (*M*_w_: from 7.5 to 47.8 kg mol^−1^) over time with a modest broadening in the distributions evident (*M*_w_/*M*_n_: from 2.1 to 6.7) (Figure 9). Interestingly, by representing this GPC data as d*N*_f_/(d log *M*) vs. log *M*_n_ plots ([Supplementary-material supplementary-material-1][Supplementary-material supplementary-material-1]), where d*N*_f_ stands for the number fraction of macromolecules having molecular weight *M*_n_, some evidence for two types of active sites was evident. In particular, close inspection of [Supplementary-material supplementary-material-1] reveals a low molecular weight polyethylene that disappeared on prolonged reaction time. This observation may suggest a minor contribution of a second type of active site that gradually reduced as the run proceeded. Nevertheless, a gradual increase of *M*_n_ with polymerization time represents the major trend which is in line with a suppression of the chain transfer rate owing to a depletion of the aluminum-alkyl chain transfer agent [13]. On lowering the ethylene pressure to 5 atm (run 17, Table 4), a decline in activity (4.17 × 10^6^ g(PE) mol^−1^ (Fe) h^−1^) was observed (run 17 vs. run 9, Table 4). Such pressure effects can be attributed to the lower solubility of ethylene in toluene at an ambient ethylene pressure as compared to that at higher pressure [11, 32, 33, 36, 43, 44].

Finally, under the optimized conditions identified for **Fe4**/MAO (Al : Fe = 1500, *T* = 60°C, *t* = 30 minutes), the performances of **Fe1**–**Fe3** and **Fe5** were also explored (runs 18-21, Table 4) and the results discussed alongside that for **Fe4**. In general, these MAO-promoted polymerizations showed high activity though less than that seen with MMAO (6.08–9.60 × 10^6^ g(PE) mol^–1^ (Fe) h^–1^ with MAO vs. 8.50–15.86 × 10^6^ g(PE) mol^−1^ (Fe) h^−1^ with MMAO) with the resulting polyethylenes displaying broad unimodal molecular weight distributions (*M*_w_/*M*_n_ = 3.8–9.3). As with **Fe1**-**Fe5/**MMAO, a similar trend in activities was apparent, **Fe4** [2,4,6-tri(Me)] > **Fe1** [2,6-di(Me)] > **Fe5** [2,6-di(Et)-4-Me] > **Fe2** [2,6-di(Et)] > **Fe3** [2,6-di(*i*-Pr)], with the least sterically bulky **Fe4** and **Fe1** showing higher activity than the more bulky comparators, **Fe2**, **Fe5**, and **Fe3**. Unlike that seen with MMAO, the *para*-methyl group in **Fe4** and **Fe5** had a positive influence on activity in a manner similar to that described elsewhere [36–38]. In addition, the polyethylene obtained with the most hindered precatalyst **Fe3** [2,6-di(*i*-Pr)] exhibited the highest molecular weight (72.6 kg mol^−1^, run 20, Table 4) [32, 36–38, 43, 44].

**Figure 10:**
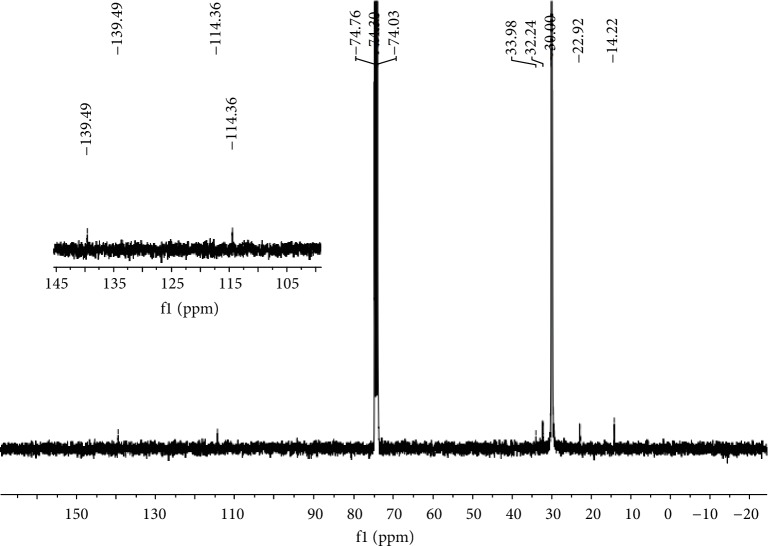
^13^C NMR spectrum of the polyethylene generated using **Fe4**/MAO at 60°C in C_2_D_2_Cl_4_ (run 9, Table 4).

**Figure 11:**
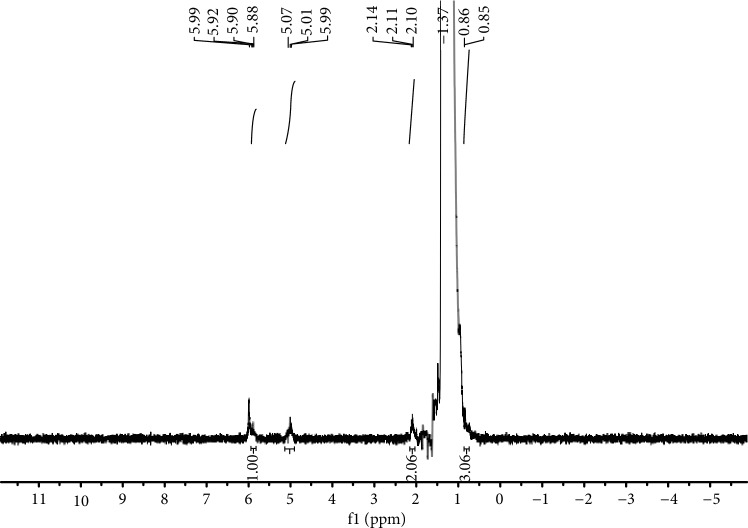
^1^H NMR spectrum of the polyethylene generated using **Fe4**/MAO at 60°C in C_2_D_2_Cl_4_ (run 9, Table 4).

With reference to the polymers generated using **Fe1**–**Fe5**/MAO, the melting temperatures were all around 130°C (Table 4) in accord with a highly linear polymeric backbone. This assertion was supported by the ^1^H and ^13^C NMR spectra of a sample of polyethylene obtained using **Fe4** at 60°C (run 9, Table 4) with a high-intensity single resonance centered around *δ* 30.0 for the methylene repeat unit in ^13^C NMR spectrum along with the corresponding peak at *δ* 1.37 in the ^1^H NMR spectrum (Figures 10 and 11) [22, 26, 27, 32, 33, 36–38]. Interestingly, the ^13^C NMR spectrum also revealed weaker vinylic carbon resonances (–CH=CH_2_) at around *δ* 114.36 and *δ* 139.49 along with more upfield *n*-propyl peaks (*δ* 14.22, 22.92, and 32.24). Support for the presence of a vinyl end-group was further provided by the appearance of downfield proton resonances at *δ* 5.01 and *δ* 5.90 in the ^1^H NMR spectrum (Figure 11). Based on these NMR observations, it would imply that the main termination pathway in this MAO-promoted polymerization involves *β*-hydride elimination [4, 11, 32, 36, 38, 43, 44].

## 3. Conclusions

In summary, a new family of iron(II) chloride complexes of type **E** (**Fe1**–**Fe5**) bound by an unsymmetrical chelating bis(imino)pyridine ligand fused with both six- and seven-membered carbocyclic rings has been successfully synthesized and fully characterized. Comparison of the structural properties of **Fe3** (6/7) with iron comparators containing the symmetrically fused *N,N,N*-ligands, **B** (6/6), **C** (7/7), and **D** (8/8), highlights the effects of variation in ring strain/flexibility as well as steric and chelation properties. Upon treatment with either MMAO or MAO, **Fe1**–**Fe5** showed exceptionally high activities (15.86 × 10^6^ g(PE) mol^−1^ (Fe) h^−1^ at 40°C) for ethylene polymerization forming strictly linear polyethylenes with a broad range of molecular weights. The steric properties of the precatalyst were shown to be influential with the least sterically hindered 2,6-Me_2_-containing **Fe1** and **Fe4** displaying higher activity than the more hindered analogues **Fe2** (R^1^ = Et), **Fe3** (R^1^ = *i*-Pr), and **Fe5** (R^1^ = Et) forming wax-like materials. By contrast, higher molecular weight polymer was obtained with the most sterically encumbered precatalyst **Fe3**. The polyethylenes were found to display distributions anywhere between narrow unimodal and broad bimodal that could be, to some degree, influenced by the nature/amount of the aluminoxane cocatalyst, temperature, and run time. Moreover, end-group analysis highlighted the role of both *β*-H elimination (vinyl chain ends) and chain transfer to aluminum (saturated chain ends) as termination pathways. Overall, these hybrid 6-/7-membered ring catalysts exhibit excellent performance characteristics in ethylene polymerization when compared with iron-based **B** (6/6), **C** (7/7), and **D** (8/8), that can, to some level, be explained in terms of the steric properties imparted by the fused carbocycles and the chelation properties of the *N,N,N*-pincer ligand.

## 4. Materials and Methods

### 4.1. General Considerations

Synthetic procedures requiring moisture/air-sensitive compounds were performed under nitrogen by using standard Schlenk techniques. Toluene, the solvent used for the polymerization runs, was heated to reflux over sodium and distilled under nitrogen prior to use. Methylaluminoxane (MAO, 1.46 M solution in toluene) and modified methylaluminoxane (MMAO, 1.93 M in *n-*heptane) were acquired from Albemarle Corporation. High-purity ethylene was purchased from Beijing Yanshan Petrochemical Co. and used as received. Other reagents were purchased from Acros, Aldrich, or local suppliers. The properties of the resulting polymeric materials such as melting temperatures (*T*_m_) were measured by differential scanning calorimetry (DSC), while the molecular weight (*M*_w_) and dispersity (*M*_w_/*M*_n_) were determined by gel permeation chromatography (GPC). In selected cases, high-temperature ^1^H and ^13^C NMR spectroscopy has also been undertaken to gain further information on the structural properties of the polyolefinic materials; gas chromatography (GC) has been employed in all cases to detect for any short-chain oligomeric fractions (e.g., C_4_–C_32_). The ^1^H and ^13^C NMR spectra of the polyethylenes were recorded with a Bruker DMX 300 MHz instrument at 135°C in C_2_D_2_Cl_4_ with TMS as internal standard. Elemental analysis was carried out with a Flash EA 1112 microanalyzer, while the IR spectra were recorded using a Perkin Elmer System 2000 Fourier-Transform infrared (FT-IR) spectrometer. Molecular weights and molecular weight distributions of the polyethylenes were determined with an Agilent PLGPC 220 GPC system at 150°C with 1,2,4-trichlorobenzene as solvent. The melting temperatures of the polyethylenes were measured from the fourth scanning run on a Perkin Elmer TA-Q2000 differential scanning calorimeter under a nitrogen atmosphere. Typically, a sample of about 5.0 mg was heated to 140°C at a rate of 20°C min^–1^, maintained for 2 min at 140°C to remove the thermal history, and then cooled to -40°C at a rate of 20°C min^–1^. The unsymmetrical diketone, 2,3,7,8,9,10-hexahydro-1H-cyclohepta[*b*]quinoline-4,6-dione, was synthesized using a previously reported procedure [38].

### 4.2. 4,6-Bis(Arylimino)-1,2,3,7,8,9,10-HeptahydroCyclohepta[*b*]-Quinoline-Iron(II) Chlorides (**Fe1**–**Fe5**)

#### 4.2.1. Aryl = 2,6-Me_2_C_6_H_3_**Fe1**

A mixture of 2,3,7,8,9,10-hexahydro-1H-cyclohepta[*b*]quinoline-4,6-dione (0.23 g, 1.0 mmol), 2,6-dimethylaniline (0.49 g, 4.0 mmol), and FeCl_2_·4H_2_O (0.19 g, 1.0 mol) in glacial CH_3_COOH (10 mL) was stirred and heated to reflux for 12 h. On cooling to room temperature, an excess of cooled Et_2_O was added to induce precipitation and the precipitate collected by filtration. The solid was dissolved in MeOH (5 mL) and the solution then concentrated on the rotary evaporator. Et_2_O (20 mL) was added to precipitate the product which was collected by filtration and dried under reduced pressure yielding **Fe1** as a blue powder (0.45 g, 80%). FT-IR (cm^−1^) values are as follows: 772 (s), 803 (w), 844 (w), 936 (w), 1057 (w), 1109 (w), 1185 (w), 1248 (w), 1361 (w), 1383 (w), 1460 (s), 1569 (w), 1605 (m, *v*_C=N_), 2866 (w), 2960 (w). Anal. Calcd for C_30_H_33_Cl_2_N_3_Fe (562.36) are as follows: H, 5.92, C, 64.07, N, 7.47; found: H, 6.03, C, 63.93, N, 7.33%.

#### 4.2.2. Aryl = 2,6-Et_2_C_6_H_3_**Fe2**

By using a similar procedure to that described for **Fe1**, **Fe2** was isolated as a blue powder (0.47 g, 76%). FT-IR (cm^−1^) values are as follows: 778 (s), 811 (m), 923 (w), 1058 (w), 1111 (w), 1188 (w), 1240 (w), 1330 (w), 1452 (s), 1608 (m, *v*_C=N_), 2873 (w), 2964 (m). Anal. Calcd for C_34_H_41_Cl_2_N_3_Fe (618.47) are as follows: H, 6.68, C, 66.30, N, 6.79; found: H, 6.76, C, 66.14, N, 6.63%.

#### 4.2.3. Aryl = 2,6-*i*-Pr_2_C_6_H_3_**Fe3**

By using a similar procedure to that described for **Fe1**, **Fe3** was isolated as a blue powder (0.46 g, 68%). FT-IR (cm^−1^) values are as follows: 697 (s), 773 (s), 827 (w), 949 (w), 1037 (w), 1091 (w), 1196 (w), 1235 (w), 1260 (w), 1378 (w), 1466 (s), 1586 (w), 1614 (m, *v*_C=N_), 2863 (w), 2943 (w). Anal. Calcd for C_38_H_49_Cl_2_N_3_Fe (674.58) are as follows: H, 7.32, C, 67.66, N, 6.23; found: H, 7.46, C, 67.54, N, 6.13%.

#### 4.2.4. Aryl = 2,4,6-Me_3_C_6_H_2_**Fe4**

By using a similar procedure to that described for **Fe1**, **Fe4** was isolated as a blue powder (0.48 g, 81%). FT-IR (cm^−1^) values are as follows: 716 (w), 737 (m), 823 (w), 854 (s), 911 (w), 953 (w), 1014 (w), 1039 (w), 1083 (w), 1154 (w), 1216 (m), 1371 (w), 1429 (s), 1536 (m), 1608 (m, C=N), 2860 (w), 2916 (w). Anal. Calcd for C_32_H_37_Cl_2_N_3_Fe (590.41) are as follows: H, 6.32, C, 65.10, N, 7.12; found: H, 6.46, C, 65.04, N, 7.05%.

#### 4.2.5. Aryl = 2,6-Et_2_-4-MeC_6_H_2_**Fe5**

By using a similar procedure to that described for **Fe1**, **Fe5** was isolated as a blue powder (0.48 g, 74%). FT-IR (cm^−1^) values are as follows: 856 (s), 949 (w), 1058 (w), 1150 (w), 1207 (w), 1262 (w), 1338 (w), 1454 (s), 1565 (w), 1604 (m, *v*_C=N_), 2870 (w), 2931 (w), 2964 (w). Anal. Calcd for C_35_H_43_Cl_2_N_3_Fe (645.23) are as follows: H, 7.02, C, 66.88, N, 6.50; found: H, 7.09, C, 66.74, N, 6.56%.

### 4.3. Polymerization Studies

#### 4.3.1. Ethylene Polymerization at P_C2H4_ = 1 atm

A 100 mL Schlenk tube, equipped with a stirrer, was employed for the lower pressure polymerization runs. Under an atmosphere of C_2_H_4_, **Fe4** (3.0 *μ*mol) was added followed by toluene (30 mL) and then the required amount of cocatalyst (MAO, MMAO) introduced by using a syringe. The solution was then stirred at 1 atm C_2_H_4_ with the temperature set at the required value. After 30 min, the pressure was released and the reaction mixture quenched with 30 mL of C_2_H_5_OH (10% HCl). The polymer was washed with C_2_H_5_OH, dried under reduced pressure at 50°C, and weighed.

#### 4.3.2. Ethylene Polymerization at P_C2H4_ = 5 or 10 atm

A 250 mL stainless steel autoclave, equipped with a mechanical stirrer, a temperature controller, and an ethylene pressure control system, was employed for the higher pressure polymerization runs (5 or 10 atm C_2_H_4_). The autoclave was evacuated and refilled with ethylene three times. Firstly, when the required temperature was reached, the selected iron complex (3 *μ*mol), dissolved in toluene (30 mL), was injected into the autoclave under an atmosphere of ethylene (*ca.* 1 atm), followed by the addition of more toluene (30 mL). Secondly, the required amount of cocatalyst (MAO and MMAO) and additional toluene were added successively by syringe taking the total volume of solvent to 100 mL. The autoclave was immediately pressurized with 5 or 10 atm ethylene, and the stirring commenced. After the required reaction time (5, 10, 30, 45, and 60 min), the reactor was cooled to room temperature with a water bath and the excess ethylene pressure vented. The reaction was quenched with 30 mL of C_2_H_5_OH (10% HCl). The polymer was collected and washed with C_2_H_5_OH and dried under reduced pressure at 50°C and weighed.

### 4.4. X-Ray Structure Determination

X-ray diffraction (XRD) was employed to determine the molecular structure of **Fe3**. The XRD patterns were conducted on a Rigaku Sealed Tube CCD (Saturn 724+) diffractometer with graphite-monochromated Mo-K*α* radiation (*λ* = 0.71073 Å) at 173(2) K, and the cell parameters were obtained by global refinement of the positions of all collected reflections. Intensities were corrected for Lorentz and polarization effects and empirical absorption. The structures were solved by direct methods and refined by full-matrix least-squares on *F*^2^. All nonhydrogen atoms were refined anisotropically, and all hydrogen atoms were placed in calculated positions. Structure solution and structure refinement were performed using SHELXT-2015 [47, 48]. Crystal data and processing parameters for **Fe3** are summarized in [Supplementary-material supplementary-material-1].

## Figures and Tables

**Table 1:** Selected bond lengths and angles for **Fe3**.

Bond lengths (Å)		Bond angles (°)	
Fe1-N1	2.060(1)	N1-Fe1-N2	74.17(7)
Fe1-N2	2.216(1)	N1-Fe1-N3	73.10(7)
Fe1-N3	2.216(1)	N1-Fe1-Cl1	150.80(6)
Fe1-Cl1	2.231(7)	N1-Fe1-Cl2	91.57(6)
Fe2-Cl2	2.308(7)	N2-Fe1-N3	140.70(7)
N1-C1	1.341(3)	N2-Fe1-Cl1	100.47(5)
N1-C9	1.334(3)	N2-Fe1-Cl2	102.41(5)
N2-C2	1.283(3)	N3-Fe1-Cl1	97.59(5)
N2–C27	1.438(3)	N3-Fe1-Cl2	99.64(5)
N3–C15	1.436(3)	Cl1-Fe1-Cl2	117.48(3)

**Table 2:** Catalytic evaluation of **Fe4**/MMAO at 1 atm C_2_H_4_*^a^*.

Run	Al : Fe	*T* (°C)	*t* (min)	Mass of PE (g)	Activity*^b^*	*M*_w_ *^c^*	*M*_w_/*M*_*n*_^c^	*T*_m_ *^d^*
1	1000	20	30	1.50	1.00	15.7	15.4	125.8
2	1500	20	30	1.82	1.21	14.7	15.0	125.5
3	2000	20	30	2.41	1.61	10.8	12.4	124.4
4	2500	20	30	2.75	1.82	6.2	8.2	121.6
5	3000	20	30	1.22	0.81	5.8	7.2	121.3
6	2500	10	30	2.45	1.63	9.5	13.5	124.4
7	2500	30	30	2.48	1.65	5.9	7.9	121.2
8	2500	40	30	1.68	1.12	1.9	2.2	121.3
9	2500	50	30	1.53	1.02	1.8	2.6	119.3
10	2500	60	30	0.60	0.40	1.2	2.2	116.8

*^a^*Conditions: 3.0 *μ*mol of **Fe4**, 30 mL of toluene, 1 atm C_2_H_4_. *^b^*Activity in units of 10^6^ g(PE) mol^−1^ (Fe) h^−1^. *^c^*Determined by GPC, *M*_w_ in units of kg mol^−1^. *^d^*Determined by DSC.

**Table 3:** Catalytic evaluation of **Fe1**–**Fe5**/MMAO at higher C_2_H_4_ pressure*^a^.*

Run	Precat.	Al : Fe	*t* (min)	*T* (°C)	Mass of PE (g)	Activity*^b^*	*M*_pk_		*M*_w_ *^c^*	*M*_w_/*M*_n_*^c^*	*T*_m_ *^d^* (°C)
							Peak 1	Peak 2			
1	**Fe4**	2500	30	30	17.56	11.70	1.2 (51%)	33.1 (49%)	59.9	16.0	129.6
2	**Fe4**	2500	30	40	22.73	15.15	2.9 (70%)	28.8 (30%)	41.42	13.4	129.1
3	**Fe4**	2500	30	50	17.85	11.93	3.1 (100%)		25.42	10.6	127.2
4	**Fe4**	2500	30	60	15.72	10.48	3.5 (100%)		18.4	7.1	126.7
5	**Fe4**	2500	30	70	12.53	8.35	4.8 (100%)		5.6	2.0	126.5
6	**Fe4**	2500	30	80	6.74	4.49	1.8 (100%)		1.9	1.5	121.2
7	**Fe4**	2000	30	40	19.25	12.33	1.4 (61%)	24.4 (39%)	35.5	11.0	129.5
8	**Fe4**	2250	30	40	20.35	13.56	1.4 (62%)	23.6 (38%)	34.3	10.4	129.2
9	**Fe4**	2750	30	40	21.95	14.63	1.8 (63%)	22.9 (37%)	27.6	7.3	129.0
10	**Fe4**	3000	30	40	19.75	13.16	1.7 (69%)	22.3 (31%)	21.7	8.6	128.4
11	**Fe4**	3250	30	40	15.35	10.23	1.1 (76%)	22.2 (24%)	16.7	7.7	128.1
12	**Fe4**	2500	5	40	9.34	37.36	2.6 (100%)		5.8	2.9	124.1
13	**Fe4**	2500	15	40	18.56	24.74	1.4 (63%)	24.4 (37%)	31.9	10.4	128.1
14	**Fe4**	2500	45	40	23.74	10.55	2.2 (58%)	31.1 (42%)	61.5	15.0	129.7
15	**Fe4**	2500	60	40	25.65	8.55	1.8 (44%)	32.1 (56%)	72.9	20.8	130.7
16*^e^*	**Fe4**	2500	30	40	12.78	8.52	1.1 (51%)	40.8 (49%)	43.8	18.3	129.3
17	**Fe1**	2500	30	40	23.74	15.86	1.7 (57%)	23.6 (43%)	47.2	13.6	129.6
18	**Fe2**	2500	30	40	18.42	12.28	1.2 (73%)	35.1 (27%)	47.8	16.3	129.8
19	**Fe3**	2500	30	40	12.75	8.50	2.8 (56%)	56.3 (44%)	65.6	24.7	130.0
20	**Fe5**	2500	30	40	15.43	10.29	1.2 (65%)	44.7 (35%)	48.1	18.2	129.9

*^a^*Conditions: 3.0 *μ*mol of **Fe1**–**Fe5**, 100 mL toluene, 10 atm C_2_H_4_. *^b^*Activity in units of 10^6^ g(PE) mol^−1^ (Fe) h^−1^. *^c^*Determined by GPC, *M*_w_ in units of kg mol^−1^. *^d^*Determined by DSC. *^e^*5 atm C_2_H_4_.

**Table 4:** Catalytic evaluation of **Fe1**-**Fe5**/MAO at higher ethylene pressure*^a^*.

Run	Precat.	Al : Fe	*t* (min)	*T* (°C)	Mass of PE (g)	Activity*^b^*	*M*_w_ *^c^*	*M*_w_/*M*_n_*^c^*	*T*_m_ *^d^* (°C)
1	**Fe4**	2000	30	30	6.28	4.18	64.2	15.3	131.3
2	**Fe4**	2000	30	40	10.65	7.11	47.7	8.3	131.4
3	**Fe4**	2000	30	50	11.78	7.85	24.6	5.1	130.0
4	**Fe4**	2000	30	60	13.78	9.28	14.2	3.8	131.0
5	**Fe4**	2000	30	70	12.96	8.64	9.5	3.5	128.0
6	**Fe4**	2000	30	80	12.63	8.42	7.8	1.7	127.3
7	**Fe4**	2000	30	90	8.16	5.44	7.5	1.6	127.9
8	**Fe4**	1250	30	60	13.20	8.80	21.8	3.1	130.1
9	**Fe4**	1500	30	60	14.44	9.60	21.4	3.8	130.8
10	**Fe4**	1750	30	60	14.02	9.34	18.0	3.7	130.1
11	**Fe4**	2250	30	60	12.15	8.10	15.6	4.0	130.0
12	**Fe4**	2500	30	60	10.76	7.17	15.9	5.1	129.8
13	**Fe4**	1500	5	60	5.36	21.44	7.5	2.1	127.7
14	**Fe4**	1500	15	60	9.89	13.06	13.7	2.7	128.8
15	**Fe4**	1500	45	60	15.45	6.87	31.1	5.8	130.6
16	**Fe4**	1500	60	60	16.18	5.39	47.8	6.7	130.9
17*^e^*	**Fe4**	1500	30	60	6.25	4.17	16.3	7.8	129.0
18	**Fe1**	1500	30	60	14.07	9.38	30.1	5.7	130.3
19	**Fe2**	1500	30	60	12.93	8.62	37.3	6.6	130.6
20	**Fe3**	1500	30	60	9.12	6.08	72.6	8.1	131.4
21	**Fe5**	1500	30	60	13.12	8.74	44.6	9.0	130.3

*^a^*Conditions: 3.0 *μ*mol of **Fe1**–**Fe5**, 100 mL toluene, 10 atm C_2_H_4_. *^b^*Activity in units of 10^6^ g(PE) mol^−1^ (Fe) h^−1^. *^c^*Determined by GPC, *M*_w_ in units of kg mol^−1^. *^d^*Determined by DSC. *^e^*5 atm C_2_H_4_.

**Table 5:** Catalytic evaluation of **Fe4**/MAO at 1 atm C_2_H_4_*^a^*.

Run	Al : Fe	*T* (°C)	*t* (min)	Mass of PE (g)	Activity^b^	*M*_w_^c^	*M*_w_/*M*_n_^c^	*T*_m_^d^
1	1000	20	30	0.71	4.73	85.4	13.7	131.4
2	1500	20	30	0.78	5.20	53.7	13.8	131.5
3	2000	20	30	0.98	6.53	46.9	20.6	129.6
4	2500	20	30	0.75	5.00	41.5	19.8	129.4
5	3000	20	30	0.72	4.80	27.6	16.2	129.6
6	2000	10	30	0.82	5.47	113.3	22.6	133.4
7	2000	30	30	1.82	12.13	77.3	22.9	131.8
8	2000	40	30	1.62	10.80	31.3	15.3	128.4
9	2000	50	30	0.32	2.13	30.6	15.6	128.7
10	2000	60	30	Trace	—	—	—	—

*^a^*Conditions: 3.0 *μ*mol of **Fe4**, 30 mL of toluene, 1 atm C_2_H_4_. *^b^*Activity in units of 10^5^ g(PE) mol^−1^ (Fe) h^−1^. *^c^*Determined by GPC, *M*_w_ in units of kg mol^−1^. *^d^*Determined by DSC.

## Data Availability

All data is available in the main text or the Supplementary Materials.

## Supplementary Materials

Supplementary Materials Table S1: polymerization screening using **A**_Me2Ph_/MMAO (rescreened in Sun lab at 10 atm). Table S2: polymerization screening using **B**_Me2Ph_/MMAO (rescreened in Sun lab at 10 atm). Table S3: polymerization screening using **C**_Me2Ph_/MMAO. Table S4: polymerization screening using **D**_Me2Ph_/MMAO. Table S5: polymerization screening using **Fe1**(**E**_Me2Ph_)/MMAO. Table S6: crystal data and structure refinement for **Fe3**. Figure S1: GPC traces (d*W*_f_/(d log *M*) vs. log *M*) for the PEs produced using **Fe4**/MMAO at different reaction times (10 atm C_2_H_4_, 40°C, and Al : Fe ratio = 2500; runs 2 and 12–15, Table 3). Figure S2: GPC traces (d*N*_f_/(d log *M*_n_) vs. log *M*_n_) for the PEs generated using **Fe4**/MAO at different reaction times (10 atm C_2_H_4_, 60°C, and Al : Fe ratio = 1500; runs 13–16, Table 4). The corresponding GPC traces, represented using d*W*_f_/(d log *M*) vs. log *M*, are shown in Figure 9.

## References

[1] Small B. L., Brookhart M., Bennett A. M. A. (1998). Highly active iron and cobalt catalysts for the polymerization of ethylene. *Journal of the American Chemical Society*.

[2] Small B. L., Brookhart M. (1998). Iron-based catalysts with exceptionally high activities and selectivities for oligomerization of ethylene to linear *α*-olefins. *Journal of the American Chemical Society*.

[3] Britovsek G. J. P., Gibson V. C., McTavish S. J. (1998). Novel olefin polymerization catalysts based on iron and cobalt. *Chemical Communications*.

[4] Britovsek G. J. P., Bruce M., Gibson V. C. (1999). Iron and cobalt ethylene polymerization catalysts bearing 2,6-bis(imino)pyridyl ligands: synthesis, structures, and polymerization studies. *Journal of the American Chemical Society*.

[5] Mitchell N. E., Long B. K. (2019). Recent advances in thermally robust, late transition metal-catalyzed olefin polymerization. *Polymer International*.

[6] Wang Z., Solan G. A., Zhang W., Sun W.-H. (2018). Carbocyclic-fused N,N,N-pincer ligands as ring-strain adjustable supports for iron and cobalt catalysts in ethylene oligo-/polymerization. *Coordination Chemistry Reviews*.

[7] Flisak Z., Sun W.-H. (2015). Progression of diiminopyridines: from single application to catalytic versatility. *ACS Catalysis*.

[8] Ma J., Feng C., Wang S. (2014). Bi- and tri-dentate imino-based iron and cobalt pre-catalysts for ethylene oligo-/polymerization. *Inorganic Chemistry Frontiers*.

[9] Zhang W., Sun W. H., Redshaw C. (2013). Tailoring iron complexes for ethylene oligomerization and/or polymerization. *Dalton Transactions*.

[10] Britovsek G. J. P., Mastroianni S., Solan G. A. (2000). Oligomerisation of ethylene by bis(imino)pyridyliron and -cobalt complexes. *Chemistry - A European Journal*.

[11] Mahmood Q., Guo J., Zhang W., Ma Y., Liang T., Sun W.-H. (2018). Concurrently improving the thermal stability and activity of ferrous precatalysts for the production of saturated/unsaturated polyethylene. *Organometallics*.

[12] Mahmood Q., Yue E., Guo J. (2018). Nitro-functionalized bis(imino)pyridylferrous chlorides as thermo-stable precatalysts for linear polyethylenes with high molecular weights. *Polymer*.

[13] Semikolenova N. V., Sun W.-H., Soshnikov I. E. (2017). Origin of “multisite-like” ethylene polymerization behavior of the single-site nonsymmetrical bis(imino)pyridine iron(II) complex in the presence of modified methylaluminoxane. *ACS Catalysis*.

[14] Guo L., Zada M., Zhang W. (2019). Highly linear polyethylenes tailored with 2,6-bis[1-(p-dibenzo-cycloheptylarylimino)ethyl]pyridylcobalt dichlorides. *Dalton Transactions*.

[15] Wang K., Wedeking K., Zuo W., Zhang D., Sun W.-H. (2008). Iron(II) and cobalt(II) complexes bearing N-((pyridin-2-yl)methylene)-quinolin-8-amine derivatives: synthesis and application to ethylene oligomerization. *Journal of Organometallic Chemistry*.

[16] Xiao L., Gao R., Zhang M., Li Y., Cao X., Sun W.-H. (2009). 2-(1H-2-Benzimidazolyl)-6-(1- (arylimino)ethyl)pyridyl iron(II) and cobalt(II) dichlorides: syntheses, characterizations, and catalytic behaviors toward ethylene reactivity. *Organometallics*.

[17] Sun W.-H., Hao P., Zhang S. (2007). Iron(II) and cobalt(II) 2-(benzimidazolyl)-6-(1-(arylimino)ethyl)pyridyl complexes as catalysts for ethylene oligomerization and polymerization. *Organometallics*.

[18] Huang Y., Zhang R., Liang T., Hu X., Solan G. A., Sun W.-H. (2019). Selectivity effects on *N,N,N*′-Cobalt catalyzed ethylene dimerization/trimerization dictated through choice of aluminoxane cocatalyst. *Organometallics*.

[19] Zhang S., Sun W.-H., Xiao T., Hao X. (2010). Ferrous and cobaltous chlorides bearing 2,8-bis(imino)quinolines: highly active catalysts for ethylene polymerization at high temperature. *Organometallics*.

[20] Wang L., Sun W.-H., Han L., Yang H., Hu Y., Jin X. (2002). Late transition metal complexes bearing 2,9-bis(imino)-1,10-phenanthrolinyl ligands: synthesis, characterization and their ethylene activity. *Journal of Organometallic Chemistry*.

[21] Sun W.-H., Jie S., Zhang S. (2006). Iron complexes bearing 2-imino-1,10-phenanthrolinyl ligands as highly active catalysts for ethylene oligomerization. *Organometallics*.

[22] Zhang W., Chai W., Sun W.-H., Hu X., Redshaw C., Hao X. (2012). 2-(1-(Arylimino)ethyl)-8-arylimino-5,6,7-trihydroquinoline Iron(II) chloride complexes: synthesis, characterization, and ethylene polymerization behavior. *Organometallics*.

[23] Sun W.-H., Kong S., Chai W. (2012). 2-(1-(Arylimino)ethyl)-8-arylimino-5,6,7-trihydroquinolylcobalt dichloride: synthesis and polyethylene wax formation. *Applied Catalysis A: General*.

[24] Ba J., Du S., Yue E., Hu X., Flisak Z., Sun W.-H. (2015). Constrained formation of 2-(1-(arylimino)ethyl)-7-arylimino-6,6-dimethylcyclopentapyridines and their cobalt(II) chloride complexes: synthesis, characterization and ethylene polymerization. *RSC Advances*.

[25] Zhang Y., Huang C., Hao X., Hu X., Sun W.-H. (2016). Accessing highly linear polyethylenes by 2-(1-aryliminoethyl)-7-arylimino-6,6-dimethylcyclopenta[b]pyridylchromium (III) chlorides. *RSC Advances*.

[26] Huang F., Xing Q., Liang T. (2014). 2-(1-Aryliminoethyl)-9-arylimino-5,6,7,8-tetrahydrocycloheptapyridyl iron(II) dichloride: synthesis, characterization, and the highly active and tunable active species in ethylene polymerization. *Dalton Transactions*.

[27] Zhang Y., Suo H., Huang F., Liang T., Hu X., Sun W.-H. (2017). Thermo-stable 2-(arylimino)benzylidene-9-arylimino-5,6,7,8-tetrahydrocyclohepta[b]pyridyliron (II) precatalysts toward ethylene polymerization and highly linear polyethylenes. *Journal of Polymer Science Part A: Polymer Chemistry*.

[28] Huang F., Zhang W., Yue E., Liang T., Hu X., Sun W.-H. (2016). Controlling the molecular weights of polyethylene waxes using the highly active precatalysts of 2-(1-aryliminoethyl)-9-arylimino-5,6,7,8-tetrahydrocycloheptapyridylcobalt chlorides: synthesis, characterization, and catalytic behavior. *Dalton Transactions*.

[29] Huang F., Zhang W., Sun Y., Hu X., Solan G. A., Sun W. H. (2016). Thermally stable and highly active cobalt precatalysts for vinyl-polyethylenes with narrow polydispersities: integrating fused-ring and imino-carbon protection into ligand design. *New Journal of Chemistry*.

[30] Guo J., Wang Z., Zhang W. (2019). Highly linear polyethylenes achieved using thermo-stable and efficient cobalt precatalysts bearing carbocyclic-fused *NNN*-pincer ligand. *Molecules*.

[31] Appukuttan V. K., Liu Y., Son B. C., Ha C.-S., Suh H., Kim I. (2011). Iron and cobalt complexes of 2,3,7,8-tetrahydroacridine-4,-5(1H,6H)-diimine sterically modulated by substituted aryl rings for the selective oligomerization to polymerization of ethylene. *Organometallics*.

[32] du S., Wang X., Zhang W., Flisak Z., Sun Y., Sun W. H. (2016). A practical ethylene polymerization for vinyl-polyethylenes: synthesis, characterization and catalytic behavior of *α*,*α*′-bisimino-2,3:5,6-bis(pentamethylene)pyridyliron chlorides. *Polymer Chemistry*.

[33] Du S., Zhang W., Yue E., Huang F., Liang T., Sun W.-H. (2016). *α*,*α*′-Bis(arylimino)-2,3:5,6-bis(pentamethylene)pyridylcobalt chlorides: synthesis, characterization, and ethylene polymerization behavior. *European Journal of Inorganic Chemistry*.

[34] Suo H., Oleynik I. I., Bariashir C. (2018). Strictly linear polyethylene using Co-catalysts chelated by fused bis(arylimino)pyridines: probing ortho-cycloalkyl ring-size effects on molecular weight. *Polymer*.

[35] Bariashir C., Wang Z., Suo H. (2019). Narrow dispersed linear polyethylene using cobalt catalysts bearing cycloheptyl-fused bis(imino)pyridines; probing the effects of ortho-benzhydryl substitution. *European Polymer Journal*.

[36] Wang Z., Zhang R., Zhang W. (2019). Enhancing thermostability of iron ethylene polymerization catalysts through *N*,*N*,*N*-chelation of doubly fused *α*,*α*′-bis(arylimino)-2,3:5,6-bis(hexamethylene) pyridines. *Catalysis Science & Technology*.

[37] Wang Z., Solan G. A., Mahmood Q. (2018). Bis(imino)pyridines incorporating doubly fused eight-membered rings as conformationally flexible supports for cobalt ethylene polymerization catalysts. *Organometallics*.

[38] Wang Z., Ma Y., Guo J. (2019). Bis(imino)pyridines fused with 6- and 7-membered carbocylic rings as *N,N,N*-scaffolds for cobalt ethylene polymerization catalysts. *Dalton Transactions*.

[39] Bariashir C., Wang Z., Du S. (2017). Cycloheptyl-fused NNO-ligands as electronically modifiable supports for M(II) (M = Co, Fe) chloride precatalysts; probing performance in ethylene oligo-/polymerization. *Journal of Polymer Science Part A: Polymer Chemistry*.

[40] Abu-Surrah A. S., Lappalainen K., Piironen U., Lehmus P., Repo T., Leskelä M. (2002). New bis(imino)pyridine-iron(II)- and cobalt(II)-based catalysts: synthesis, characterization and activity towards polymerization of ethylene. *Journal of Organometallic Chemistry*.

[41] Sun W.-H., Tang X., Gao T., Wu B., Zhang W., Ma H. (2004). Synthesis, characterization, and ethylene oligomerization and polymerization of ferrous and cobaltous 2-(ethylcarboxylato)-6-iminopyridyl complexes. *Organometallics*.

[42] Xiao T., Hao P., Kehr G., Hao X., Erker G., Sun W.-H. (2011). Dichlorocobalt(II) complexes ligated by bidentate 8-(benzoimidazol-2-yl)quinolines: synthesis, characterization, and catalytic behavior toward ethylene. *Organometallics*.

[43] Chen Q., Zhang W., Solan G. A., Liang T., Sun W.-H. (2018). Methylene-bridged bimetallic bis(imino)pyridine-cobaltous chlorides as precatalysts for vinyl-terminated polyethylene waxes. *Dalton Transactions*.

[44] Chen Q., Zhang W., Solan G. A. (2018). CH(phenol)-bridged bis(imino)pyridines as compartmental supports for diiron precatalysts for ethylene polymerization: exploring cooperative effects on performance. *Organometallics*.

[45] Jones D. J., Gibson V. C., Green S. M., Maddox P. J., White A. J. P., Williams D. J. (2005). Discovery and optimization of new chromium catalysts for ethylene oligomerization and polymerization aided by high-throughput screening. *Journal of the American Chemical Society*.

[46] Tomov A. K., Gibson V. C., Britovsek G. J. P. (2009). Distinguishing chain growth mechanisms in metal-catalyzed olefin oligomerization and polymerization systems: C_2_H_4_/C_2_D_4_ co-oligomerization/ polymerization experiments using chromium, iron, and cobalt catalysts. *Organometallics*.

[47] Sheldrick G. M. (2015). SHELXT-integrated space-group and crystal structure determination. *Acta Crystallographica Section A Foundations and Advances*.

[48] Sheldrick G. M. (2015). Crystal structure refinement with SHELXL. *Acta Crystallographica Section C Structural Chemistry*.

