# Supplementary material for: Fusing Carbocycles of Inequivalent Ring Size to a Bis(imino)pyridine-Iron Ethylene Polymerization Catalyst: Distinctive Effects on Activity, PE Molecular Weight, and Dispersity
Source: Research (Wash D C). 2019 Oct 16;2019:9426063. doi: 10.34133/2019/9426063 (PMC6946285; doi:10.34133/2019/9426063)
Supplement: Supplementary Materials — Table S1: polymerization screening using AMe2Ph/MMAO (rescreened in Sun lab at 10 atm). Table S2: polymerization screening using BMe2Ph/MMAO (rescreened in Sun lab at 10 atm). Table S3: polymerization screening using CMe2Ph/MMAO. Table S4: polymerization screening using DMe2Ph/MMAO. Table S5: polymerization screening using Fe1(EMe2Ph)/MMAO. Table S6: crystal data and structure refinement for Fe3. Figure S1: GPC traces (dWf/(d log M) vs. log M) for the PEs produced using Fe4/MMAO at different reaction times (10 atm C2H4, 40°C, and Al : Fe ratio = 2500; runs 2 and 12–15, Table 3). Figure S2: GPC traces (dNf/(d log Mn) vs. log Mn) for the PEs generated using Fe4/MAO at different reaction times (10 atm C2H4, 60°C, and Al : Fe ratio = 1500; runs 13–16, Table 4). The corresponding GPC traces, represented using dWf/(d log M) vs. log M, are shown in Figure 9. [file 9426063.f1.doc]

**Supplementary Material**

for

Fusing carbocycles of inequivalent ring size to a bis(imino)pyridine-iron ethylene polymerization catalyst; distinctive effects on activity, PE molecular weight and dispersity

Zheng Wang,^a,b^ Gregory A. Solan,^a,c^,* Yanping Ma,^a,b^ Qingbin Liu,^d^* Tongling Liang^a^ and Wen-Hua Sun^a,b,e^*

^a^ Key Laboratory of Engineering Plastics and Beijing National Laboratory for Molecular Science, Institute of Chemistry, Chinese Academy of Sciences, Beijing 100190, China.

^b^ CAS Research/Education Center for Excellence in Molecular Sciences, University of Chinese Academy of Sciences, Beijing 100049, China.

^c^ Department of Chemistry, University of Leicester, University Road, Leicester LE1 7RH, UK.

^d^ College of Chemistry and Material Science, Hebei Normal University, Shijiazhuang 050024, China.

^e^ State Key Laboratory for Oxo Synthesis and Selective Oxidation, Lanzhou Institute of Chemical Physics, Chinese Academy of Sciences, Lanzhou 730000, China.

*** Corresponding Authors**

E-mail: [whsun@iccas.ac.cn](mailto:whsun@iccas.ac.cn); Tel: +86-10-62557955; Fax: +86-10-62618239 (W.-H.S).

E-mail: [gas8@leicester.ac.uk](mailto:gas8@leicester.ac.uk); Tel: +44-116-2522096 (G.A.S.)

E-mail: [qbinliu@sina.com](mailto:qbinliu@sina.com)；Tel: +86-0311-80787432 (Q. L).

| **Table of Contents** | | Page |
| --- | --- | --- |
| 1. | General considerations | S2 |
| 2. | Catalytic performance data for **A**_Me2Ph_, **B**_Me2Ph_, **C**_Me2Ph_, **D**_Me2Ph_ and **E**_Me2Ph_ (**Fe1**); all runs conducted using MMAO, *P*_C2H4_ = 10 atm | S3 |
| 3. | GPC traces for the PEs produced | S4 |
| 4. | X-ray structure determination | S5 |
| 5. | References | S5 |

**1. General considerations**

All the synthetic procedures involving air- and moisture-sensitive compounds were carried out under a nitrogen atmosphere by using standard Schlenk techniques. Toluene was heated to reflux over sodium and distilled under nitrogen prior to use in the polymerization runs. Methylaluminoxane (MAO, 1.46 M solution in toluene) and modified methylaluminoxane (MMAO, 1.93 M in *n-*heptane) were purchased from Albemarle Corporation. High-purity (99.9%) ethylene was purchased from Beijing Yansan Petrochemical Co. and used as received. Other reagents were purchased from Acros, Aldrich or local suppliers. NMR spectra were recorded with a Bruker DMX 400 MHz instrument at ambient temperature by using TMS as internal standard. Molecular weights (*M*_w_) and molecular weight distributions (*M*_w_/*M*_n_) of the polyethylenes were determined with an Agilent PLGPC 220 GPC system at 150 °C with 1,2,4-trichlorobenzene as solvent. IR spectra were recorded with a Perkin Elmer System 2000 FTIR spectrometer. Elemental analysis was carried out with a Flash EA 1112 microanalyzer. The melting temperatures of the polyethylenes were measured from the fourth scanning run on a Perkin Elmer TA-Q2000 differential scanning calorimeter under a nitrogen atmosphere. A sample of about 5.0 mg was heated to 140 °C at a rate of 20 °C min^–1^, kept for 2 min at 140 °C to remove the thermal history, and then cooled to -40 °C at a rate of 20 °C min^–1^. The ^13^C NMR spectra of the polyethylenes were recorded with a Bruker DMX 300 MHz instrument at 135 °C in C_2_D_2_Cl_4_. 2,3,7,8,9,10- Hexahydro-1H-cyclohepta[b]quinoline-4,6-dione was prepared according to the literature procedure.[73] X-ray crystallographic studies data. CCDC 1908415 (**Fe3**) contain the supplementary crystallographic data for this paper. These data can be obtained free of charge via www.ccdc.cam.ac.uk/data_request/cif, or by emailing data_request@ccdc.cam.ac.uk, or by contacting the Cambridge Crystallographic Data Centre, 12 Union Road, Cambridge CB2 1EZ, UK; fax: +44 1223 336033.

**2.** **Catalytic performance data for A_Me2Ph_, B_Me2Ph_, C_Me2Ph_, D_Me2Ph_ and E_Me2Ph_ (Fe1); all runs conducted using MMAO, *P*_C2H4_ = 10 atm**

| **Table S1** Polymerization screening using **A**_Me2Ph_/MMAO (re-screened in Sun lab at 10 atm) | | | | | | | | |
| --- | --- | --- | --- | --- | --- | --- | --- | --- |
| Run | Al:Fe | T (^o^C) | t (min) | Mass of PE (g) | Activity*^b^* | *M*_w_*^c^* | *M*_w_/*M*_n_*^c^* | *T*_m_ (^o^C)*^d^* |
| 1 | 2500 | 40 | 30 | 7.26 | 4.84 | 34.4 | 10.7 | 129.5 |
| 2 | 2500 | 50 | 30 | 16.24 | 10.83 | 18.7 | 8.4 | 128.3 |
| ^a^ Conditions: 3.0 μmol of **A**_Me2Ph_, 100 mL toluene, 10 atm C_2_H_4_.  ^b^ Values in units of 10^6^ g(PE) mol^-1^ (Fe) h^-1^.  ^c^ Determined by GPC, and *M*_w_ in kg mol^−1^.  ^d^ Determined by DSC. | | | | | | | | |

| **Table S2** Polymerization screening using **B**_Me2Ph_/MMAO (re-screened in Sun lab at 10 atm) | | | | | | | | |
| --- | --- | --- | --- | --- | --- | --- | --- | --- |
| Run | Al:Fe | T (^o^C) | t (min) | Mass of PE (g) | Activity*^b^* | *M*_w_*^c^* | *M*_w_/*M*_n_*^c^* | *T*_m_ (^o^C)*^d^* |
| 1 | 2500 | 40 | 30 | 7.80 | 5.20 | 7.6 | 4.5 | 125.9 |
| 2 | 2500 | 50 | 30 | 15.12 | 10.08 | 6.1 | 3.7 | 124.4 |
| ^a^ Conditions: 3.0 μmol of **B**_Me2Ph_, 100 mL toluene, 10 atm C_2_H_4_.  ^b^ Values in units of 10^6^ g(PE) mol^-1^ (Fe) h^-1^.  ^c^ Determined by GPC, and *M*_w_ in kg mol^−1^.  ^d^ Determined by DSC | | | | | | | | |

| **Table S3** Polymerization screening using **C**_Me2Ph_/MMAO | | | | | | | | | |
| --- | --- | --- | --- | --- | --- | --- | --- | --- | --- |
| Run | Al:Fe | T (^o^C) | t (min) | Mass of PE (g) | Activity*^b^* | *M*_w_*^c^* | *M*_w_/*M*_n_*^c^* | *T*_m_ (^o^C)*^d^* | |
| 1 | 1800 | 40 | 30 | 11.40 | 7.60 | 82.8 | 26.8 | | 131.5 |
| 2 | 1800 | 50 | 30 | 15.93 | 10.62 | 54.1 | 22.0 | | 129.6 |
| *^a^* Conditions: 3.0 μmol of **C**_Me2Ph_, 100 mL toluene, 10 atm C_2_H_4_.  *^b^* Values in units of 10^6^ g(PE) mol^-1^ (Fe) h^-1^.  *^c^* Determined by GPC and *M*_w_ in kg mol^−1^.  *^d^* Determined by DSC | | | | | | | | | |

| **Table S4** Polymerization screening using **D**_Me2Ph_/MMAO | | | | | | | | | |
| --- | --- | --- | --- | --- | --- | --- | --- | --- | --- |
| Run | Al:Fe | T (^o^C) | t (min) | Mass of PE (g) | Activity*^b^* | | *M*_w_*^c^* | *M*_w_/*M*_n_*^c^* | *T*_m_ (^o^C)*^d^* |
| 1 | 2000 | 40 | 30 | 15.88 | 10.59 | | 147.7 | 28.6 | 131.6 |
| 2 | 2000 | 50 | 30 | 18.34 | 12.23 | | 133.7 | 34.1 | 131.1 |
| *^a^* Conditions: 3.0 μmol of **D**_Me2Ph_, 100 mL toluene, 10 atm C_2_H_4_.  *^b^* Values in units of 10^6^ g(PE) mol^-1^ (Fe) h^-1^.  *^c^* Determined by GPC, and *M*_w_ in kg mol^−1^.  *^d^* Determined by DSC. | | | | | | | | | |
| **Table S5** Polymerization screening using **Fe1**(**E**_Me2Ph_)/MMAO | | | | | | | | | |
| Run | Al:Fe | T (^o^C) | t (min) | Mass of PE (g) | | Activity*^b^* | *M*_w_*^c^* | *M*_w_/*M*_n_*^c^* | *T*_m_ (^o^C)*^d^* |
| 1 | 2500 | 40 | 30 | 23.74 | | 15.86 | 47.2 | 13.6 | 129.6 |
| 2 | 2500 | 50 | 30 | 22.35 | | 12.65 | 32.4 | 10.8 | 129.0 |
| *^a^* Conditions: 3.0 μmol of **Fe1 (E**_Me2Ph_), 100 mL toluene, 10 atm C_2_H_4_.  *^b^* Values in units of 10^6^ g(PE) mol^-1^ (Fe) h^-1^.  *^c^* Determined by GPC and *M*_w_ in kg mol^−1^.  *^d^* Determined by DSC | | | | | | | | | |

**3. GPC traces for the PEs generated using Fe4/MMAO at different reaction times**


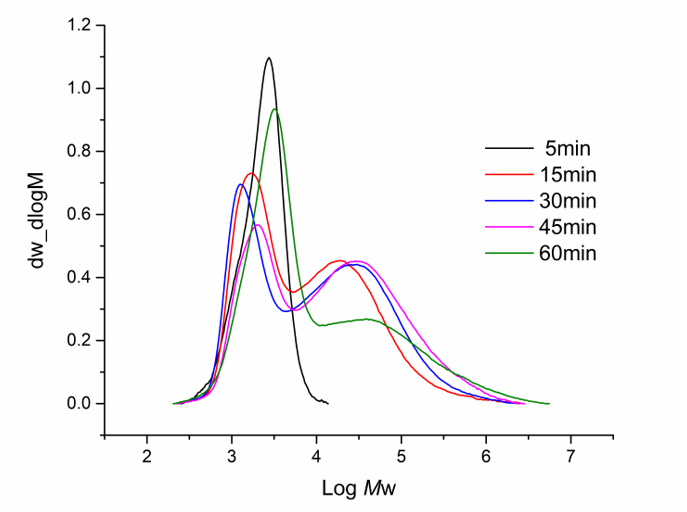


**Figure S1** GPC traces (d*W*_f_/(d log *M*) *vs.* log *M*) for the PEs produced using **Fe4**/MMAO at different reaction times (10 atm C_2_H_4_, 40 ^o^C and Al:Fe ratio = 2500; runs 2 and 12 – 15, Table 3)


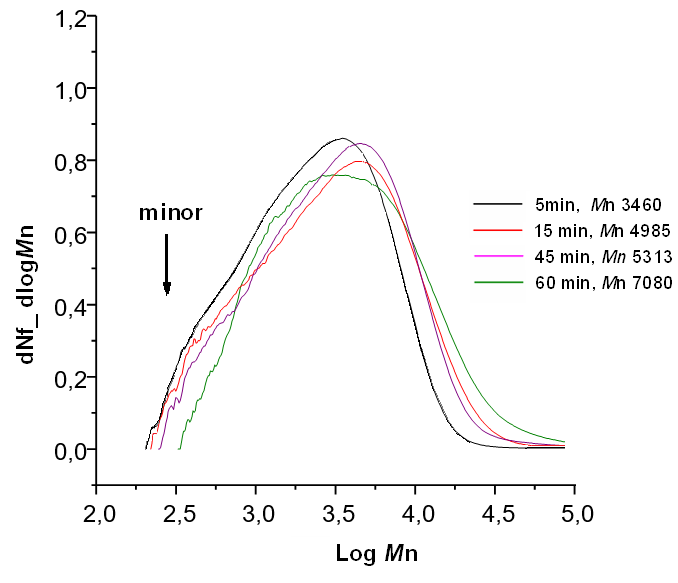


**Figure S2** GPC traces (d*N*_f_/(d log *M*_n_) *vs.* log *M*_n_) for the PEs generated using **Fe4**/MAO at different reaction times (10 atm C_2_H_4_, 60 ^o^C and Al:Fe ratio = 1500; runs 13 – 16, Table 5). Note: d*N*_f_ stands for the number fraction of macromolecules possessing molecular weight *M*.[6] The corresponding GPC traces, represented using d*W*_f_/(d log *M*) *vs.* log *M*, are shown
in Figure 8.

**4**. X-ray structure determination

| **Table S6** Crystal data and structure refinement for **Fe3** | |
| --- | --- |
| Identification code | **Fe3** |
| CCDC | 1908415 |
| Empirical formula | C_38_H_49_Cl_2_FeN_3_ |
| Formula weight | 674.55 |
| Temperature/K | 170.00(10) |
| Crystal system | monoclinic |
| Space group | P2_1_/n |
| a/Å | 10.1091(3) |
| b/Å | 24.9573(8) |
| c/Å | 14.2035(4) |
| α/° | 90 |
| β/° | 95.199(3) |
| γ/° | 90 |
| Volume/Å^3^ | 3568.74(19) |
| Z | 4 |
| ρ_calc_g/cm^3^ | 1.255 |
| μ/mm^‑1^ | 0.602 |
| F(000) | 1432.0 |
| Crystal size/mm^3^ | 0.258 × 0.172 × 0.061 |
| Radiation | MoKα (λ = 0.71073) |
| 2Θ range for data collection/° | 6.93 to 62.066 |
| Index ranges | -13 ≤ h ≤ 14, -35 ≤ k ≤ 30, -19 ≤ l ≤ 18 |
| Reflections collected | 37645 |
| Independent reflections | 9911 [R_int_= 0.0404, R_sigma_ = 0.0455] |
| Data/restraints/parameters | 9911/0/405 |
| Goodness-of-fit on F^2^ | 1.038 |
| Final R indexes [I>=2σ (I)] | R_1_ = 0.0563, wR_2_ = 0.1397 |
| Final R indexes [all data] | R_1_ = 0.0824, wR_2_ = 0.1522 |
| Largest diff. peak/hole / e Å^-3^ | 1.72/-0.56 |

**5. References**

1. Z. Wang, Y. Ma, J. Guo, Q. Liu, Y. Sun, G. A. Solan, T. Liang, and W.-H. Sun, “Bis(imino)pyridines fused with 6- and 7-membered carbocylic rings as *N,N,N*-scaffolds for cobalt ethylene polymerization catalysts,” *Dalton Trans.,* **2019***,* 48, 2582-2591.
2. G. J. P. Britovsek, M. Bruce, V. C. Gibson, B. S. Kimberley, P. J. Maddox, S. Mastroianni, S. J. McTavish, C. Redshaw, G. A. Solan, S. Strömberg, A. J. P. White, and D. J. Williams, “Iron and cobalt ethylene polymerization catalysts bearing 2,6-bis(Imino)pyridyl ligands: synthesis, structures, and polymerization studies,” *J. Am. Chem. Soc*., **1999**, *121*, 8728–8740.
3. V. K. Appukuttan, Y. Liu, B. C. Son, C. -S. Ha, H. Suh, I. Kim, “Iron and cobalt complexes of 2,3,7,8-tetrahydroacridine-4,-5(1H,6H)-diimine sterically modulated by substituted aryl rings for the selective oligomerization to polymerization of ethylene,” *Organometallics*, **2011**, *30*, 2285–2294.
4. S. Du, X. Wang, W. Zhang, Z. Flisak, Y. Sun, W.-H. Sun, “A practical ethylene polymerization for vinyl-polyethylenes: synthesis, characterization and catalytic behavior of α,α’-bisimino-2,3:5,6-bis- (pentamethylene)pyridyliron chlorides,” *Polym. Chem*., **2016**, 7, 4188–4197.
5. Z. Wang, R. Zhang, G. A. Solan, W. Zhang, Q. Liu, T. Liang, and W.-H. Sun, “Enhancing thermostability of iron ethylene polymerization catalysts through N,N,N-chelation of doubly fused α,α′-bis(arylimino)-2,3:5,6-bis(hexamethylene) pyridines,” *Catal. Sci. Technol.*, **2019**, 9, 1933–1943.

[6] N. V. Semikolenova, W.-H. Sun, I. E. Soshnikov, M. A. Matsko, O. V. Kolesova, V. A. Zakharov and K. P. Bryliakov, “Origin of “multisite-like” ethylene polymerization behavior of the single-site nonsymmetrical bis(imino)pyridine iron(II) complex in the presence of modified methylaluminoxane,” *ACS Catal*., **2017**, 7, 2868-2877.
